# Implications of a Change of Paradigm in Alpha1 Antitrypsin Deficiency Augmentation Therapy: From Biochemical to Clinical Efficacy

**DOI:** 10.3390/jcm9082526

**Published:** 2020-08-05

**Authors:** José Luis López-Campos, Laura Carrasco Hernandez, Candelaria Caballero Eraso

**Affiliations:** 1 Unidad Médico-Quirúrgica de Enfermedades Respiratorias, Instituto de Biomedicina de Sevilla (IBiS), Hospital Universitario Virgen del Rocío/Universidad de Sevilla, 41013 Seville, Spain; lauracarrascohdez@gmail.com (L.C.H.); ccaballero-ibis@us.es (C.C.E.); 2 Centro de Investigación Biomédica en Red de Enfermedades Respiratorias (CIBERES), Instituto de Salud Carlos III, 28029 Madrid, Spain

**Keywords:** alpha1 antitrypsin deficiency, augmentation therapy, replacement therapy, rare diseases

## Abstract

Ever since the first studies, restoring proteinase imbalance in the lung has traditionally been considered as the main goal of alpha1 antitrypsin (AAT) replacement therapy. This strategy was therefore based on ensuring biochemical efficacy, identifying a protection threshold, and evaluating different dosage regimens. Subsequently, the publication of the results of the main clinical trials showing a decrease in the progression of pulmonary emphysema has led to a debate over a possible change in the main objective of treatment, from biochemical efficacy to clinical efficacy in terms of lung densitometry deterioration prevention. This new paradigm has produced a series controversies and unanswered questions which face clinicians managing AAT deficiency. In this review, the concepts that led to the approval of AAT replacement therapy are reviewed and discussed under a new prism of achieving clinical efficacy, with the reduction of lung deterioration as the main objective. Here, we propose the use of current knowledge and clinical experience to face existing challenges in different clinical scenarios, in order to help clinicians in decision-making, increase interest in the disease, and stimulate research in this field.

## 1. Introduction

Alpha1 antitrypsin deficiency (AATD) is a rare genetic condition that determines the appearance of pulmonary emphysema and liver damage in its severe forms [1]. As a rare condition, the available evidence indicating how to proceed in different clinical situations that may occur is limited and not always clear-cut. Therefore, controversies and doubts may arise about the management of different aspects of this condition in daily clinical practice. This is particularly evident when it comes to considering exogenous alpha1 antitrypsin (AAT) replacement therapy, which is also known as augmentation therapy, for severely deficient patients. Different aspects including indications, the dose regimen for dose intervals, or the differences between the presentations available are still a matter for debate. Interestingly, in recent years, there have been some advances that have clarified, at least in part, some of the previous controversies in relation to this therapy. One major finding is the ability of AAT augmentation therapy to decrease emphysema progression as measured by lung densitometry [2,3,4]. Consequently, the preservation of pulmonary density with exogenous AAT has sparked a debate over a potential change in the main aims of treatment from biochemical efficacy to clinical efficacy, based on emphysema progression evaluated by lung densitometry. Under this new paradigm, clinicians managing AATD are faced with a number of controversies and unanswered questions. In this work, we propose harnessing current knowledge and clinical experience to find answers to the current debate, with a view to helping clinicians to take key decisions, arouse interest in the disease, and foster research in this field.

## 2. Initial Assumptions

### 2.1. Biochemical Efficacy

**Figure 1:**
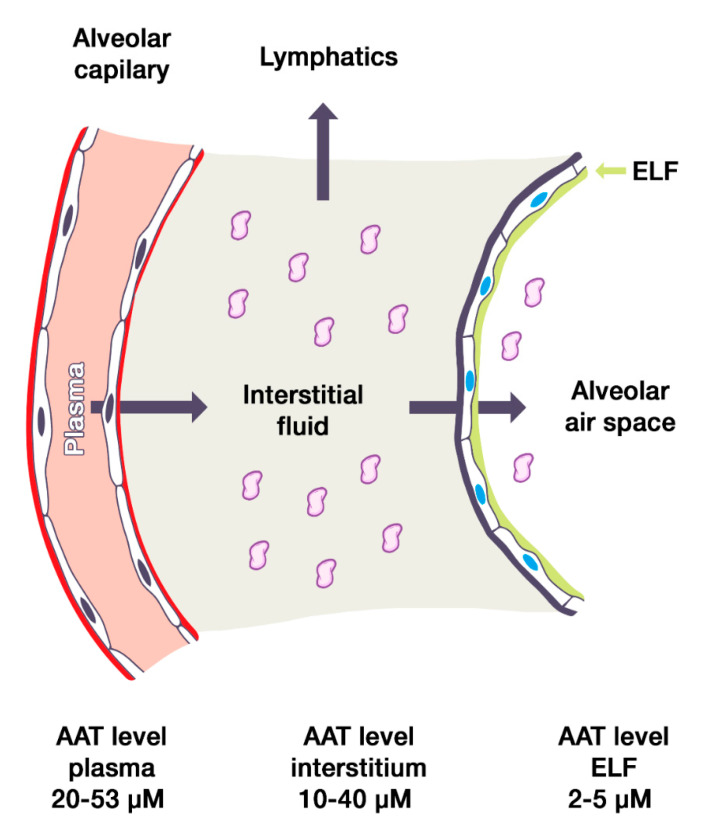
Diagram indicating alpha1 antitrypsin in plasma from normal individuals diffusing across the alveolar capillary endothelial barrier into the interstitium. AAT, Alpha1 antitrypsin; ELF, epithelial lining fluid. The sizes of the vascular, interstitial, and alveolar compartments are not to scale, for educational purposes.

Plasma AAT normally diffuses through the endothelial barrier into the interstitium. Here, most of the AAT flows out through the lymphatic vessels; however, part of the diffuse AAT passes through the epithelial barrier into the alveolar epithelial lining fluid (Figure 1). Accordingly, the amount of serum AAT is directly proportional to the amount of AAT that migrates to the interstitium and the epithelial lining fluid of the lungs [5]. As a result, the initial trials on AAT replacement therapy were focused on demonstrating that this therapy could restore the AAT levels and anti-elastase activity in the epithelial lining fluid. This aim is referred to as biochemical efficacy.

**Figure 2:**
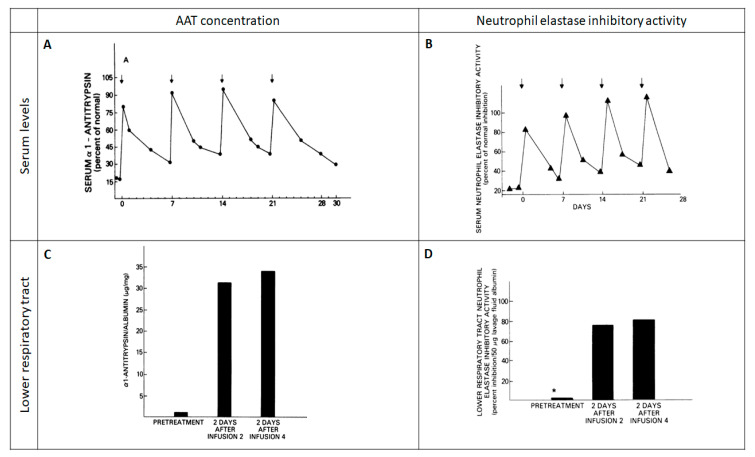
Initial study evaluating the biochemical efficacy of AAT augmentation therapy. Reproduced with permission from Reference [6]. (**A**) The response of serum al-antitrypsin levels to the infusion of 4.0 g of AAT. (**B**) Serum neutrophil elastase inhibitory activity following weekly intravenous infusions of 4.0 g of AAT. (**C**) Lower respiratory tract al-antitrypsin levels during intravenous replacement therapy with 4.0 g of AAT. (**D**) Lower respiratory tract neutrophil elastase inhibitory activity following weekly intravenous infusions of 4.0 g of AAT.

However, four figures obtained from five patients (Figure 2) in 1981 [6] were enough to start a path that would culminate in the approval of the Food and Drug Administration in 1987. This initial study demonstrated that, by administering intravenous exogenous AAT in seven-day infusion periods, exogenous AAT could reach the epithelial lining fluid, increase the protein concentration at this compartment, and improve the anti-elastase activity [7]. A subsequent study in a few patients also confirmed that, by administering AAT infusions every seven days, serum levels of AAT were kept above a level defined as a protective threshold (discussed below) [8].

Finally, AAT replacement therapy was approved for intravenous infusion based on biochemical efficacy and preliminary safety data. Therefore, it is important to remember that, at those times, augmentation therapy for AATD was an example of a treatment that had been approved for use in patients, without being evaluated for efficacy and safety in cohorts of patients through traditional clinical trials in clinical, functional, or radiological terms.

### 2.2. Protective Threshold Level

A so-called protective threshold level has been defined as the amount of AAT in serum from which there is no increased risk of developing emphysema. Notably, this limit was not based on specific patient data but on epidemiological data, following the description of the presence of emphysema in the initial cases [8]. Interestingly, this threshold level was first described by radial immunodiffusion, which suggests that that AAT levels ≤50 mg/dL are associated with a high risk for the development of emphysema, levels between 50 and 80 mg/dL confer an uncertain risk, and levels >80 mg/dL confer no increase in risk above the background risk [8]. Therefore, 80 mg/dL by radial immunodiffusion was the initial accepted limit.

However, there is a considerable confusion regarding this level. In those days, commercially available standards yielded values for amounts of AAT that were identified as higher than the true values [8]. Therefore, the result had to be adjusted depending on which standard was used—the commercially available standard or a true laboratory standard. To differentiate the procedures, initial studies expressed values as milligrams per deciliter when based on the commercial standard, and as micromolar units when based on the true laboratory standard. According to these different units, 80 mg/dL with the commercial standard corresponded to 11.2 µM with the true laboratory standard [8]. Later, in another study, 80 mg/dL by radial immunodiffusion corresponded to 10.9 µM [9]. Consequently, in the ATS consensus, it was established that 80 mg/dL by radial immunodiffusion corresponded to 50 mg/dL by nephelometry, using commercially available standards, which in turn corresponded to 11 µM, using the American National Health Lung Blood Institute (NHLBI) standard [10]. Interestingly, 11 µM of the NHLBI standard corresponds to 57.2 mg/dL by nephelometry. Therefore, these two values of 11 µM and 57.2 mg/dL by nephelometry, using the NHBLI standard, have been interchangeably identified as the protective threshold.

Interestingly, what this protective threshold really shows is a level from which emphysema associated with the deficit is more likely to appear, based on epidemiological studies. However, we really have no idea if this is the limit above which the progression of emphysema is slowed down with replacement therapy, as the concept of “protective threshold” would imply. Accordingly, we still do not know the threshold which identifies the optimal therapeutic response in terms of emphysema progression [11], and so, rather than “protective threshold”, it would be better to call it “detection threshold” or “severity threshold” or “risk threshold”.

### 2.3. Dose

Since AAT half-life in healthy MM individuals is reported to be from 3.8 to 5.2 days (mean 4.6, standard error of the mean 0.21) [12], the initial trials devised an infusion schedule that would allow once-weekly administrations for PiZ individuals, thus maintaining serum AAT levels above the considered protective level (as discussed above). Weekly infusions containing around 4.0 g of AAT were therefore prepared from 3.5–4.0 L of pooled plasma [6]. This dose corresponds to 57.1 mg/kg for a subject of 70 kg. Accordingly, the subsequent trial used the standard dose of 60 mg/kg in weekly infusions [8], which has been maintained until now.

### 2.4. Route of Administration

Since the first studies, the administration of AAT has been intravenous [6,7,8]. This route of administration is the only one available to this day. Inhaled AAT administration has been tried in recent years, but it is still under exploration [13].

### 2.5. Origin of AAT

From the beginning, the AAT has been obtained from fresh plasma from human donors. Specifically, the first studies reported that between 3 and 4 L of pooled human plasma was necessary to obtain 4 gr of AAT for administration [6]. Alternative ways of obtaining AAT were investigated in the early years, in particular from recombinant DNA in microorganisms [14] or from recombinant AAT associated with virus vectors as a potential tool for the gene therapy [15], cell lines producing AAT [16], silkworm larvae [17], or a plant-made recombinant AAT from Nicotiana benthamiana, a close relative of the tobacco plant which is indigenous to Australia [17]. All of these are exploratory possibilities, but, currently, AAT continues to be obtained from human plasma.

### 2.6. Final Statement

Starting from the uncertain origins commented above, replacement treatment with exogenous AAT obtained from human plasma, at a dose of 60 mg/kg in weekly intravenous administrations, is the maintenance therapy for severe AATD, with the objective of achieving biochemical efficacy by aiming for serum levels of AAT above 11 µM, using the NHBLI standard.

## 3. Clinical Efficacy: A Change of Paradigm

It was only from the late 1990s onward when longitudinal data on the impact of AAT replacement therapy started to become available. This information comes mainly from seven major observational studies (Table 1), and mostly from two major cohorts, namely the German scientific working group for the therapy of lung diseases (WALT from the German abbreviation: Wissenschaftliche Arbeitsgemeinschaft zur Therapie von Lungenerkrankungen) [18,19,20] and the American NHBLI registry for individuals with severe deficiency of AAT [21,22], together with other studies in the USA [23] and Spain [24]. Additionally, three clinical trials plus one pooled analysis of two of these trials explored the clinical impact of AAT augmentation therapy [2,3,4,25] (Table 1). To cut a long story short, although observational trials reported several significant results favoring augmentation therapy in terms of exacerbations, lung function, or survival, the main finding from the formal clinical trials was a reduction in the loss of lung density by lung densitometry. This finding was relevant since it implied that, by administering intravenous exogenous AAT, not only are we able to regain biochemical efficacy, but the treatment also achieves a change in the natural progression of emphysema, with a potential longer-term prognostic impact [26,27]. Therefore, augmentation therapy has the potential to modify the natural progression of AATD-associated emphysema.

As a main consequence, the objective of exogenous administration of AAT is to be modified from seeking biochemical efficacy to seeking clinical impact in the form of prevention of lung deterioration and emphysema progression. However, this change in therapeutic objective poses a dilemma. If the goal of treatment is no longer to ensure biochemical efficacy but to slow the progression of emphysema, this opens up a number of controversies and unanswered questions as regards when the therapy is indicated and its administration and follow-up, which are discussed hereafter.

## 4. Controversies over Indication

### 4.1. Age Limits

The eligibility criteria regarding age are not very explicit in all trials. When available, these are adult studies (>18 years), and the average age is around 45 to 50 years (Table 1). Although there may be extreme values in the distributions, there is little information on very young or very old cases. Although cases in children requiring augmentation therapy have been described anecdotally [28], being young is not normally an issue from a clinical perspective, since by the time emphysema develops, patients are already young adults [29,30]. The issue comes with elderly cases. Currently, there is no information on the impact of AAT augmentation therapy in this cohort. These patients are clearly disease survivors, and the dilemma of initiating a life-long weekly intravenous treatment over the age of 70 or 80 is controversial. In these cases, despite the fact that we could restore AAT anti-elastase activity, achieving the biochemical efficacy, it is not well established how much further the emphysema may progress and what window of opportunity is available to have an impact on long-term lung density deterioration in these elderly patients. Therefore, therapy ought to be individualized and must be agreed with the patient and the caregivers, since many comorbidities and social circumstances may influence this decision. In any case, family screening should be carried out in these cases, to detect severe cases amongst younger relatives. Fortunately, current technologies facilitate these diagnostic procedures in the population [31,32].

### 4.2. Mutations

AATD is a co-dominant autosomal inherited genetic disease caused by mutations in the SERPINA1 gene on chromosome 14. Mutations are named with a letter from A to Z, according to the migration speed in the isoelectric focusing gel. The normal M allele has an average migration rate. The two most frequent mutated alleles are S and the more severe Z. As shown in Table 1, most studies include ZZ and rare or null allele carriers, with only a minority with SZ or other less frequent mutations. Interestingly, in these trials, the eligibility criteria focused on having a severe AATD (identified by an AAT value below the protective threshold), irrespective of the mutation. Although we can restore the biochemical efficacy of the approved dose for all mutations, the impact on emphysema progression has been shown for those with AAT ≤ 11 µM, which included ZZ, Zn, and other unspecified severe mutations. Accordingly, the drug′s technical specifications for augmentation therapy states that the indication is for PiZZ, PiZ (null), Pi (null) (null), and PiSZ. However, in the main study showing a clear impact on lung density, the RAPID trial [4], there were only 12 cases (7%) with severe mutations other than ZZ, which were two SZ, one Z/Null, and nine other non-specified mutations. Unfortunately, these mutations were not evaluated separately and there is no sensitivity analysis evaluating this in the trial. As a result, when aiming at biochemical efficacy, it is reasonable to consider treatment in those with severe deficits (AAT ≤ 11 µM), regardless of the associated mutation. However, the exact impact on the decline of lung densitometry in non-ZZ patients needs to be clarified in the future.

### 4.3. Lung Function Limits

The evaluation of lung function impairment in chronic obstructive pulmonary disease (COPD) is performed with FEV1. Consequently, the limits of lung function impairment in previous studies was evaluated by this parameter (Table 1). Additionally to the potential relevance of AAT in COPD [33], according to these studies, there were two ways of evaluating FEV1 as the baseline value or as the yearly decline. Although the majority of the cases had an FEV1 below 50%, the included patients presented a wide variability in this spirometric value. Interestingly, the main trial evaluating AAT augmentation therapy on densitometry, the RAPID trial, included patients with FEV1 between 35 and 70% [4]. In fact, the technical specifications for Prolastin include a formal indication of FEV1 limits from 35 to 60%. This has led to the question about whether milder or more severe cases should receive augmentation therapy to impact on emphysema progression. There are two points worth considering about this issue.

First, despite this formal lack of evidence, common sense and clinical experience urge us to indicate or not to stop augmentation therapy below an FEV1 of 35%. The clinical practice guidelines from the Alpha1 Foundation clearly state that intravenous augmentation therapy is recommended for individuals with a predicted FEV1 less than 30%, and, for those with an FEV1 greater than 65%, they recommend discussing with each individual the potential benefits of reducing lung function decline, considering the cost of the therapy and lack of evidence for its benefits [34]. Similarly, the Portuguese guidelines state that augmentation therapy should not be discontinued in case of pulmonary function deterioration, even if it reaches the lowest established limit for its initiation [35].

Second, despite the well-known role of FEV1 in COPD, we know that FEV1 is a poor surrogate measure for emphysema. Notably, gas transfer is reduced much earlier than FEV1 [36,37], and it is therefore a more sensitive marker of disease onset. Additionally, gas transfer keeps worsening in the final stages of the disease, whereas FEV1 decline slows down [38], thus making it also a more sensitive marker of disease deterioration than FEV1 [36]. Finally, during disease progression, gas transfer is a better marker of emphysema progression than FEV1. Altogether, gas transfer may be a more sensitive and specific test of emphysema development than FEV1. However, the indication for augmentation therapy continues to be established according to FEV1 values instead of diffusing capacity. Thus, if emphysema is the goal of treatment rather than biochemical efficacy, it would probably be desirable to use diffusing capacity instead of FEV1 as the basis for indication for therapy and as a monitoring and follow-up parameter. If this is the case, we should consider the FEV1 debate to be over and turn our focus to the functional parameters with a closer relationship to emphysema.

### 4.4. Indication in Liver Disease

**Figure 3:**
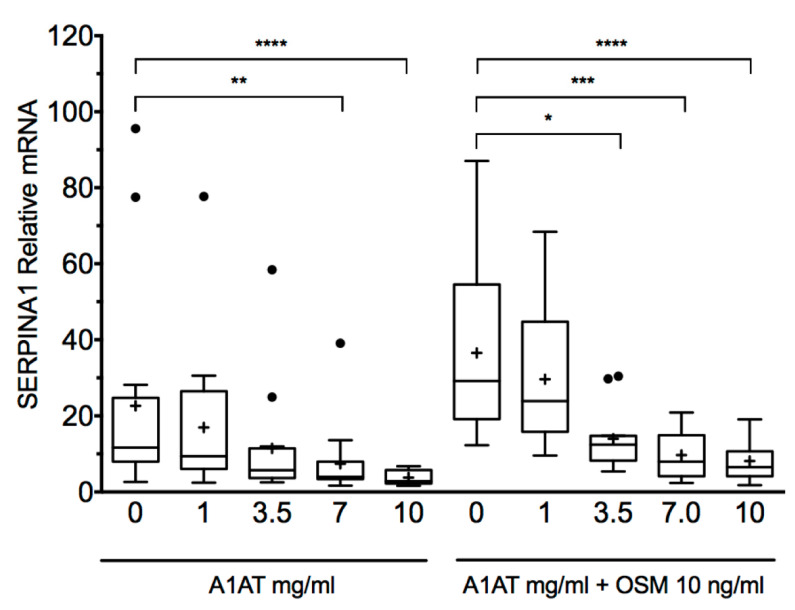
Purified A1AT reduces SERPINA1 expression in a dose-dependent manner in primary human hepatocytes. Exogenously added purified AAT reduced SERPINA1 expression in primary human hepatocytes isolated from both proficient and deficient liver tissue. The reduction was more prominent following Oncostatin M (OSM 10 ng/mL) stimulation, known to increase expression of SERPINA1. *, *P* < 0.05; **, *P* < 0.001; ***, *P* < 0.001; ****, *P* < 0.0001). © 2017 Karadagi, et al. This is an open-access article distributed under the terms of the Creative Commons Attribution License, which permits unrestricted use, distribution, and reproduction in any medium, provided the original author and source are credited. Obtained from Reference [44].

Despite the potential clinical importance of AATD-associated liver failure, the available treatments are still at the very early stages of clinical development. Currently, liver transplantation remains the definitive option for its treatment [39]. However, according to the current records, AATD is a rare cause of liver transplantation [40]. Some treatments are beginning to be evaluated, such as glibenclamide analogs [41], ursodeoxycholic acid homologues [42], or RNA-targeted treatments [43]. In this context, AAT augmentation therapy has not been explored as a potential treatment for AATD-associated liver condition until very recently. Interestingly, a recent collaborative study between Sweden and Germany reported that exogenous AAT lowered SERPINA1 expression in primary human hepatocytes in a dose-dependent manner [44] (Figure 3). Consequently, if the reproducibility and clinical relevance of these findings are confirmed, a possible new indication is opened as a treatment for the prevention of liver involvement that should be prospectively evaluated in trials with patients at risk of developing this complication.

## 5. Controversies in the Administration

### 5.1. Infusion Frequency

As discussed above, the weekly frequency of AAT infusions was based on the half-life of this protein and the biochemical efficacy of the first preparations. However, in our search for more comfortable dosages, other alternatives have been tried by doubling, tripling, and even quadrupling the dose and the administration interval to up to 240 mg/kg every four weeks. From the point of view of biochemical efficacy, it seems that administrations every three and every four weeks would be insufficient to keep the trough pre-dose value (C_min_) above the considered protection threshold (as discussed above) [45]. For this reason, it is recommended to use the usual weekly dose, which, in some cases, can be changed to a biweekly administration with double doses, since this regimen has also been shown to be appropriate [46] and safe [47]. However, if we include in the equation the objective of slowing down the progression of emphysema instead of achieving biochemical efficacy, the scenario changes considerably. Interestingly, the so-called Danish–Dutch study, the first study to evaluate the role of replacement therapy in lung density, used a dose of 250 mg/kg every four weeks, resulting in an improvement in the progression of emphysema [3]. Unfortunately, the study of lung density was an exploratory objective in which the densitometric technique was not standardized. Consequently, it would be desirable to replicate the study with the current lung densitometry methodology. Interestingly, the result of the Danish–Dutch study opens up another question about how it is possible that administration every four weeks does not maintain biochemical efficacy during the four weeks [45] but does have an effect on lung densitometry [3]. This paradox highlights the discrepancy between biochemical and clinical efficacy, which should be explored in future trials. Additionally, it supports the idea mentioned above that the so-called “protective threshold” is a misnomer and should rather be known as “the risk threshold for developing emphysema”.

### 5.2. Correct Dose

As discussed above, the weekly dose of 60 mg/kg was established following the experience of the first researchers, and it has remained to this day, since it ensured biochemical efficacy. However, the RAPID trial identified that lung density decrease rate was related with the median serum AAT concentration, so that the higher the serum concentration, the higher impact on lung density decline prevention. This data also suggested that there was no ceiling effect with the data provided by the RAPID trial [4], implying that achieving higher serum level might have a greater impact on lung density decline. This hypothesis has been recently explored in the SPARK study, which evaluated the safety and pharmacokinetic profile of weekly infusions of a 120 mg/kg dose and showed that this dose is safe and well-tolerated and provides more favorable physiologic AAT serum levels [48]. SPARTA (Study of ProlAstin-C Randomized Therapy with Alpha-1 augmentation), an ongoing randomized, placebo-controlled trial is currently assessing the efficacy and safety of 120 mg/kg administered weekly over three years [11]. The recruitment for this trial is now over, and the results, once available, will have to be evaluated to complete this discussion between the discrepancy between biochemical and clinical efficacy.

### 5.3. Differences between Preparations

**Figure 4:**
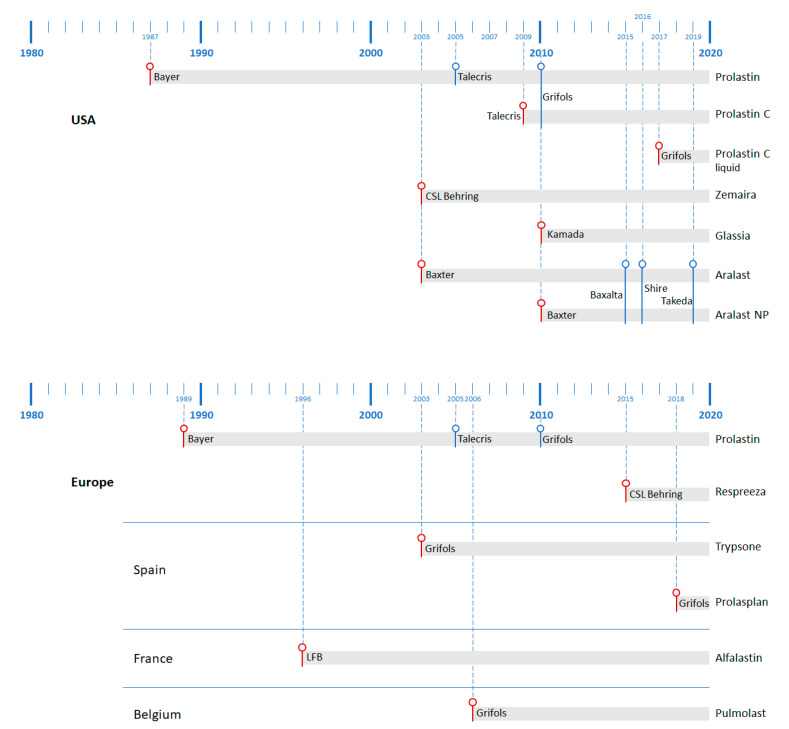
Temporal distribution of the years of approval of augmentative AAT treatments in the USA (upper) and Europe and European countries, with their own presentation (lower). In red: year of initial official approval. In blue: year when company changed. LFB, Laboratoire français du fractionnement et des biotechnologies.

**Figure 5:**
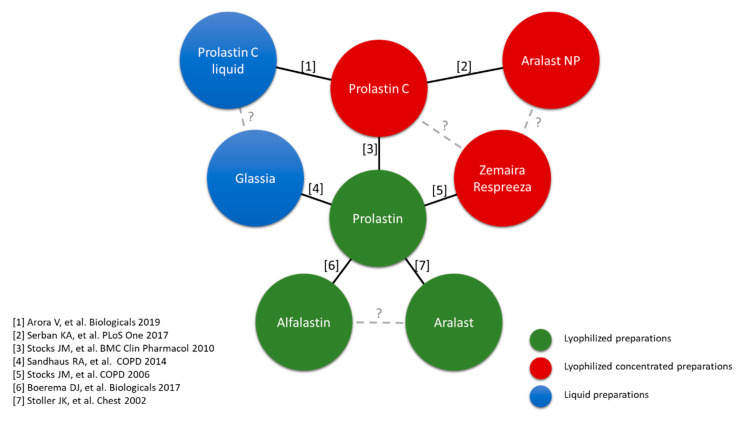
List of studies evaluating biochemical efficacy by type of preparation.

Augmentation therapy with AAT has undergone extensive pharmacological development since the first approval of Prolastin in 1987 (Figure 4). All the preparations can be divided into three types: lyophilized preparations (Prolastin and other country-specific brand names, Trypsone, Alfalastin, and Aralast, originally named Respitin), lyophilized concentrated preparations (Zemaira in the USA—known as Respreeza in Europe—Prolastin C, and Aralast NP), and finally the preparations with a liquid presentation (Prolastin C liquid and Glassia). From a biochemical efficacy perspective, all of these gained approval by comparing their clinical efficacy with either Prolastin, as the first approved presentation, or Prolastin C (pivotal studies showing biochemical efficacy are depicted in Figure 5).

However, if the aim of the treatment is to decrease loss of lung density rather than biochemical efficacy, then only three of these products have been evaluated in clinical trials, namely Alfalastin in the Danish–Dutch study [3], Prolastin in the EXACTLE (EXAcerbations and Computed Tomography scan as Lung End-points) trial [2], and Zemaira/Respreeza in the RAPID trial [4]. Nevertheless, neither the standard methodology of lung densitometry nor the approved explored dose was followed in the Danish–Dutch study. Therefore, it would be of interest to compare the differences between Prolastin and Zemaira/Respreeza in trials.

To begin with, there are certain differences between these two presentations [49]. First, it has been reported in biochemical comparisons between both that Zemaira/Respreeza shows a better profile in terms of total protein content, AAT potency, specific AAT activity, and purity [50,51]. Secondly, the concentration of AAT is different in both preparations with Zemaira/Respreeza having a higher concentration (Table 2). Thirdly, the results of the clinical trials are probably not directly comparable due to the different population and study design (Table 2). Therefore, until the possible emergence of new therapeutic options, augmentative treatment with AAT is the only specific treatment for patients with congenital emphysema, and both products have demonstrated their efficacy in slowing the progression of emphysema (Table 2). Accordingly, this treatment should be available for patients who meet internationally established criteria and are controlled and supervised by reference health centers [49]. Unfortunately, we will probably not be able to directly explore these differences further, since future trials are being planned with lyophilized concentrated or liquid preparations.

## 6. Controversies in the Follow-Up

### 6.1. Monitoring Control in the First Few Months

Due to the current controversy over dosage therapy and replacement therapy, once indicated, the next question is how to establish the suitable dose. From the perspective of biochemical efficacy, the debate is about the use of C_min_ during the initial administration as a way of identifying which patients can receive biweekly infusions. Here, some authors warn against systematic measurements of C_min_ [52], whereas others suggest there is scope for an individualized dosage regimen for AAT augmentation therapy [53]. Interestingly, if we are aiming for individualized biochemical efficacy, it follows that measuring C_min_ during the first infusions is the only way to identify how patients respond to other regimens than weekly administrations. Once established, there is no further need to evaluate it. However, if we are aiming to achieve clinical efficacy in terms of preventing lung density decline progression, we will have to admit that further information is needed. We have the results of the Danish–Dutch study, which quadrupled the dose and the administration interval and showed a significant impact on the outcome (with the limitations mentioned above) [3], despite biochemical efficacy not being guaranteed [45]. Additionally, the really interesting measure would be the evaluation of the progression of emphysema by lung densitometry. Unfortunately, the methodology used in the RAPID trial for lung density evaluation is not available for clinical practice. Alternatively, other forms of evaluation emphysema progression should be evaluated in the future to identify lung function or radiological parameters that allow us to identify the correct dose for emphysema progression prevention in the follow-up.

### 6.2. Discontinuation of Augmentation Therapy

**Figure 6:**
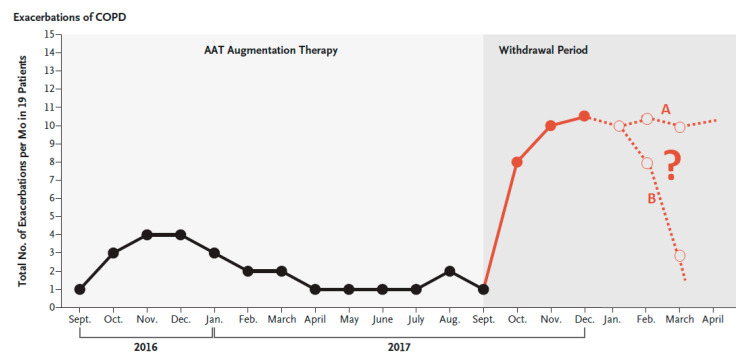
Total number of pulmonary exacerbations per month in the 19 patients in Ireland receiving AAT augmentation therapy for AAT deficiency–associated emphysema during the year before the study (black line) and during the withdrawal period (red line), as reported in the original article. Reproduced with permission from Reference [54]. On the right, we have added (dashed line) two possible hypothetical changes that the curve could have had if the patients had been followed longer, with two possibilities: curve A, with a persistent increase of exacerbations risk, and curve B, with a transient increase in the exacerbation risk. COPD, chronic obstructive pulmonary disease.

The impact of discontinuation of augmentative treatment has not been a major topic of debate in recent years until the recent publication of two letters. McElvaney, et al. [54] recently reported on the consequences of abrupt cessation of AAT replacement therapy, showing an increase in the frequency of exacerbations over a 77-day period, with two deaths due to exacerbation (Figure 6), accompanied by increased circulating levels of inflammatory biomarkers. Recently, another study [55] described post-transplant complications leading to reduced survival only in patients who had discontinued replacement therapy a few weeks before transplant. Interestingly, the effect seemed to have faded by 11 months, suggesting a possible role of the timing of the withdrawal of replacement therapy. These authors [55] hypothesized about a transient inflammatory rebound phenomenon short after withdrawal of therapy. Interestingly, the study by McElvaney, et al. [54] describes this increase in the inflammatory load during the weeks after withdrawal, which seems to peak at three months (Figure 6). The progression of the inflammatory markers and the clinical consequences beyond that point should be explored, to define the window of risk, since two behaviors could potentially be seen thereafter (Figure 6). As a result, these two letters have opened up new questions about the repercussion of abrupt cessation of augmentation therapy that should be further explored. Very recently the role of AAT as a modulator of the neutrophil membrane has been described, providing new data on the role of neutrophil-associated AATD disease [56].

### 6.3. Pulmonary Transplantation

The relationship between augmentation therapy and lung transplantation has sparked another major controversy. Beyond the debate on the survival benefits of lung transplantation in AATD [57,58,59], the issue at hand now is the role of augmentation therapy in the peritransplantation. At this point, the debate is largely mediated by the decision over whether to continue replacement therapy after transplantation, with two conflicting opinions with their own arguments (Table 3). On the one hand, augmentation therapy has been shown to have several potential benefits. First, intravenous AAT inhibits elastase mediated injury to the transplanted lung in humans [60]. Second, animal models have shown that the administration of human AAT before reperfusion in recipients improved immediate post-transplant lung function [61]. Third, AAT attenuates acute lung allograft injury [62]. On the other hand, as expected, augmentation therapy retains its biochemical efficacy in the recipient, although we do not know about the potential preventive effect of augmentation therapy on newly developed emphysema in the transplanted lung. With the available information, this decision is especially relevant, in the light of the study by Kleinerova, et al. [55] mentioned above, in which the authors suggested that discontinuation of augmentation therapy should be undertaken several months before lung transplantation, to avoid an increased risk of complications. Therefore, either augmentation therapy is stopped just at inclusion in the lung-transplant waiting list or is maintained non-stop during the complete process. Trials are needed to clarify this confusing situation.

## 7. The Future: A Second Change of Paradigm Coming

Despite the fact that the diagnostic approach to AATD currently varies between different health institutions and countries [63], diagnosis currently begins with the determination of AAT levels in blood as the main form of screening for the disease. Although this approach is probably the most suitable at the moment, it leaves us with some unknowns. It has been reported a variable disease penetrance of AATD, with different patients with the same mutations suffering different degrees of disease burden. Of note, AAT levels do not clarify why some patients develop severe lung disease, while others do not carry the same mutation [64,65]. In this context, the potential role of evaluating anti-elastase activity could be of help. Anti-elastase activity is one of the main markers in the importance of a specific mutation [66]. Beyond serum AAT levels, anti-elastase activity is also influenced by external and environmental factors like alcohol intake [67] or active smoking [68], as well as by inorganic compounds [69] and inflammatory processes [70]. In human-immunodeficiency virus patients, AAT levels increased in bronchoalveolar lavage fluid and blood. However, anti-elastase activity decreased in bronchoalveolar lavage from human-immunodeficiency virus patients, suggesting impaired AAT function [71]. Accordingly, it has been described that there is a disagreement between protein concentration in peripheral blood with anti-elastase activity [72]. Finally, to complicate things further, a mutation has been described which is secreted at normal levels in cellular models of AATD but with a severe reduction in anti-elastase activity, therefore identifying the first pure functionally deficient AATD mutation [73].

Consequently, we may need to address the debate about the adequacy of basing AATD screening exclusively on the peripheral blood concentration of AAT or if it is possible to determine anti-elastase activity as a complementary marker of lung involvement in AATD [74,75]. Beyond making general recommendations for a healthy lifestyle, which is also necessary, anti-elastase activity should probably be measured to provide a clearer idea of the potential penetration of the disease and enable us to give more thorough patient-based diagnosis and information on the presence and importance of the disease in specific cases in order to evaluate its impact on the long-term deterioration of lung density. Here, multinational clinical research collaboration initiatives [76] will pave the way for acquiring more thorough knowledge on AATD that will result in a more personalized approach for these patients.

## Figures and Tables

**Table 1:** Description of the population included in the main studies with augmentation therapy.

Study	*N*	Age		FEV1		Mutations	
		Eligibility Criteria	Cohort Results *	Eligibility Criteria	Cohort Result *	Eligibility Criteria	Cohort Result
Observational studies with control							
Seersholm N, et al.; WALT. Eur Respir J, 1997 [18]	295	NR	46 (8)	FEV1 < 65% or decline > 120 mL/year	37 (14)%	Severe AATD (NR)	NR
NHLBI Registry. AJRCCM, 1998 [21]	927	≥18 years	46 (11)	NR	<35%: 43.6% 35–49%: 21.1% 50–79%: 16.2% ≥80%: 19.1%	AAT ≤ 11 µM or ZZ	NR
Wencker M, et al.; WALT. Eur Respir J, 1998 [19]	443	>18 years	45 (7)	FEV1 < 65% or decline > 120 mL/year	Ex-smokers: 35.5 (14.8)% Non-smokers: 42.2 (18.2)%	AAT < 50 mg/dL (nephelometry) AAT < 80 mg/dL (immunodiffusion)	ZZ: 88.9% SZ: 7.1% Other/unknown: 4.0%
Wencker M, et al.; WALT. Chest, 2001 [20]	96	NR	44 (8)	FEV1 < 65% or decline > 120 mL/year	41 (17.3)%	AAT < 35%	ZZ: 85% SZ: 8% Other: 3%
Stoller JK, et al.; NHLBI. Chest, 2003 [22]	747	NR	48 (9)	NR	37 (18)%	AAT ≤ 11 µM or ZZ or null	NR
Tonelli AR, et al. Int J COPD, 2009 [23]	164	NR	61.3 (0.7)	NR	43 (2)%	ZZ	ZZ
Barros-Tizón JC, et al. Ther Adv Respir Dis, 2012 [24]	127	>18 years	51.7 (9.1)	NR	1.25 (0.50) L	AAT ≤ 11 µM (50 mg/dL) and ZZ. rare or null	ZZ: 93.6% SZ: 0.8% Other: 5.6%
Clinical trials							
Dirksen A, et al.; Danish–Dutch study. AJRCCM, 1999 [3]	56	NR	Danish: 50.4 (1.62) Dutch 45.1 (1.17)	FEV1 30–80%	Danish: 1.5 (0.9) L Dutch: 1.6 (0.1) L	ZZ	ZZ
Dirksen A, et al.; EXACTLE. Eur Respir J, 2009 [2]	77	≥18 years	54.7 (8.4)	NR	46.3 (19.6)%	AAT ≤ 11 µM	ZZ or Zn
Chapman KR, et al.; RAPID. Lancet, 2015 [4]	180	18–65	53.8 (6.9)	FEV1 35–70%	47.4 (12.1)%	AAT ≤ 11 µM	ZZ: 93% Other: 7%

* Numerical data expressed as mean (standard deviation). FEV1: forced expiratory volume in 1 s (expressed as absolute values in liters or as percentage of the theoretical value the patient should have according to its age, gender, height, weight, and race). NR, not reported; AAT, alpha1 antitrypsin ; AATD, Alpha1 antitrypsin deficiency; NHLBI, National Health Lung Blood Institute; COPD, chronic obstructive pulmonary disease; WALT, from the German abbreviation: Wissenschaftliche Arbeitsgemeinschaft zur Therapie von Lungenerkrankungen; AJRCCM, American Journal of Respiratory and Critical Care Medicine

**Table 2:** Differences between Prolastin and Zemaira/Respreeza.

	Prolastin	Zemaira/Respreeza
Origin *	Human donor plasma	Human donor plasma
Presentations *	1 gr + 40 mL solvent (25 mg/mL)	1 gr + 20 mL solvent (50 mg/mL) 4 gr + 76 mL solvent (50 mg/mL) 5 gr + 95 mL solvent (50 mg/mL)
Excipients *	Powder: Sodium chloride Sodium dihydrogen phosphate Solvent: Water for injections	Powder: Sodium chloride Sodium dihydrogen phosphate monohydrate Mannitol Solvent: Water for injections
Purity (AAT/proteins) [49]	76.9%	97.4%
Specific activity (active AAT/proteins) [49]	64%	86.2%
Infusion velocity *	0.08 mL/kg/min	0.08 mL/kg/min
Time of infusion 60 mg/kg dose †	30 min	15 min
Time of infusion 120 mg/kg dose ‡	60 min	30 min
Lung density decline reduction [4,25]	Versus basal: −1.73 g/L/year Versus placebo: 1.01 g/L/year	Versus basal: −1.45 g/L/year Versus placebo: 0.74 g/L/year

*, Obtained from the technical leaflet; †, At a dose of 60 mg/kg/wk for a 75 kg patient; ‡, At a dose of 120 mg/kg/2 wk for a 75 kg patient.

**Table 3:** Arguments for and against maintaining augmentation therapy after lung transplant.

In Favor	Against
The biochemical efficacy is expected to be the same as in non-transplanted AATD patients.	There are no formal trials on its clinical efficacy in lung density deterioration after transplant.
Augmentation therapy is safe and well-tolerated, and patients get used to it as part of their lives. It is not expected to create an additional burden.	Lung transplant patients already have to cope with a considerable amount of medication with potential adverse effects that determine their lives, without adding another treatment of unproven efficacy in this context.
AATD lung-transplant patients are generally younger, with a longer life expectancy, so it is vital to take all the necessary measures to protect the transplanted lung.	Emphysema due to AATD is a slow, progressive disease. It may take decades until clinically relevant emphysema is developed in the new lung.
The number of lung donors is limited, so every transplant has an opportunity cost, since it could have been received by another patient. Therefore, it is unethical not to take all possible steps to preserve the transplanted lung.	It has not been proven that the risk of rejection is increased if recipients do not receive augmentation therapy.
